# An EEG Database and Its Initial Benchmark Emotion Classification Performance

**DOI:** 10.1155/2020/8303465

**Published:** 2020-08-03

**Authors:** Ayan Seal, Puthi Prem Nivesh Reddy, Pingali Chaithanya, Arramada Meghana, Kamireddy Jahnavi, Ondrej Krejcar, Radovan Hudak

**Affiliations:** ^1^PDPM Indian Institute of Information Technology, Design and Manufacturing, Jabalpur 482005, India; ^2^Faculty of Informatics and Management, Center for Basic and Applied Research, University of Hradec Kralove, Rokitanskeho 62, Hradec Kralove, 50003, Czech Republic; ^3^Malaysia Japan International Institute of Technology, Universiti Teknologi Malaysia, Jalan Sultan Yahya Petra, 54100 Kuala Lumpur, Malaysia; ^4^Department of Biomedical Engineering and Measurement, Faculty of Mechanical Engineering, Technical University of Kosice, Letna 904200 Kosice, Slovakia

## Abstract

Human emotion recognition has been a major field of research in the last decades owing to its noteworthy academic and industrial applications. However, most of the state-of-the-art methods identified emotions after analyzing facial images. Emotion recognition using electroencephalogram (EEG) signals has got less attention. However, the advantage of using EEG signals is that it can capture real emotion. However, very few EEG signals databases are publicly available for affective computing. In this work, we present a database consisting of EEG signals of 44 volunteers. Twenty-three out of forty-four are females. A 32 channels CLARITY EEG traveler sensor is used to record four emotional states namely, happy, fear, sad, and neutral of subjects by showing 12 videos. So, 3 video files are devoted to each emotion. Participants are mapped with the emotion that they had felt after watching each video. The recorded EEG signals are considered further to classify four types of emotions based on discrete wavelet transform and extreme learning machine (ELM) for reporting the initial benchmark classification performance. The ELM algorithm is used for channel selection followed by subband selection. The proposed method performs the best when features are captured from the gamma subband of the FP1-F7 channel with 94.72% accuracy. The presented database would be available to the researchers for affective recognition applications.

## 1. Introduction

**Figure 1:**
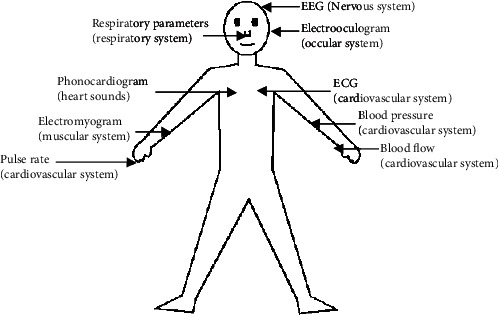
Sources of physiological signals [18].

Emotion is a mental condition [1] of a person. It is directly or indirectly connected to high intensity and high hedonic content namely, thoughts, feelings, behavioral responses, and a level of happiness or unhappiness [2]. The objective of emotion recognition is to identify a human's temporal emotional state based on facial expressions and verbal expressions automatically. Multimedia elements such as texts, audio, and video are also used frequently to induce human expressions for emotion recognition [3]. Body movements with gestures and speech are also combined with facial expressions to classify emotion more accurately [4]. Unavoidably, feelings play a significant role in making relations with others. It also helps to increase interaction with computers. Affective computing is a branch of study, where human interaction with computers using emotion and its applications are taught [5]. Affective computing finds its applications spanning in various areas like medicine, e-learning, monitoring, marketing, entertainment, and law. One can find some real-life examples of affective computing such as counseling and determining client's medical state, determining patients sentiment and relief level about the treatment during healthcare, analyzing the emotion and thereby adjusting the learning technique and presentation according to the style of a learner in the case of e-learning, remote health checkup of senior citizens, expanding means of communication and assisting the people with autism, and determining fatigue in the case of driving and alerting in advance. Since the emotional state of a human may affect attentiveness, problem-solving, and policy-making skills, the vision of affective computing is to build systems that are capable to identify and influence a person's emotion to increase yield and effectiveness of working with computers. Electroencephalography (EEG) is an electrophysiological checking strategy to record the electrical activity of the human brain. It is ordinarily noninvasive, with the electrodes placed along the scalp. EEG estimates voltage variations because of ionic current within the neurons of the cerebrum. The state-of-the-art emotion recognition methods [6, 7] can be broadly divided into three types namely, knowledge-based methods, statistical approaches, and hybrid techniques [8]. In knowledge-based, emotion detection is carried out by utilizing domain understanding and the semantic and syntactic features of language [9] using WordNet, SenticNet [10], ConceptNet, and EmotiNet [11]. However, it is unable to handle concept nuances and complex linguistic rules [8]. In statistical techniques, generally different supervised machine learning algorithms such as Support Vector Machines [12], Naive Bayes, and Maximum Entropy [13] are considered in emotion classification using a large set of annotated data. The classification accuracy is higher in the case of statistical methods, and it depends on the sufficiently large training set. Few works consider both knowledge-based techniques and statistical methods in emotion identification by exploiting complementary features [14, 15]. The classification accuracy provided by hybrid approaches is higher as compared to the previous two methods. However, it is computationally more challenging [9]. Nowadays, researchers are using physiological signals such as EEG and electrocardiogram (ECG) signals for emotion recognition [16, 17]. The different sources of physiological signals in the human body are shown in Figure 1.

However, when compared to emotion portrayed through facial expressions, emotion captured through EEG signals is more reliable because someone can fake his/her facial expressions; in other words, he/she may feel a different emotion from within and act and express something else, or in some cases, he/she might not be able to express through gestures or facial expressions the feeling they are experiencing inside. Interested readers can go through [19] for knowing other advantages of EEG signals over facial expression in emotion recognition. Data is an integral component of any emotion recognition task. It is rare to get publicly available annotated data which is essential for training and testing of machine learning algorithms. Some of the publicly available multimodal sources of data in the form of multimedia contents such as texts, audio, videos, or physiological signals to perform emotion recognition research are briefly described as follows: Belfast facial expression database [20] was created mainly for the investigation of sex differences, cultural differences, and individual differences in the expression and perception of a wide range of emotions using short TV programs and interview recordings [20]. HUMAINE facial expressions database [21] consists of almost all audio-visual recordings in diverse emotion-rich scenarios. SEMAINE is also an audio-visual recording [22] between a person and a virtual assistant and comprises of annotated emotion like angry, happy, fear, disgust, sadness, contempt, and amusement. IEMOCAP is also audio-visual recordings of dyadic sessions between actors and consists of emotion annotations such as happiness, anger, sadness, frustration, and neutral state [23]. eNTERFACE considers pleasure, irritation, sorrow, amazement, disgust, and panic in the form of audio-visual recordings from seven nationalities [24]. DEAP [16] and DREAMER [17] databases consist of EEG, ECG, and face video recordings [16]. This database is built, while people were watching film clips. All the data are annotated properly in terms of valence, arousal, and dominance of people. In [25], Alakus et al. offered an EEG database called GAMEEMO for various games.

It is clear from the literature that a few publicly available EEG signals databases exist. So, the salient contributions of the proposed study are as follows:

- A new database is presented, which consists of four emotions namely, happy, sad, neutral, and fear in the form of EEG signals of 44 subjects
- The captured EEG signals are classified based on discrete wavelet transform (DWT) and extreme learning machine (ELM).
- The ELM algorithm is used for channel selection followed by subband selection

The rest of the work is as follows: the background study related to the human brain and emotion is discussed in Section 2.  Section 3 presents a new database for affective computing. Materials and methods used for noise removal, feature extraction, and classifier are discussed in Section 4.  Section 5 presents the experimental results. Finally, the conclusion is drawn in Section 6.

## 2. Background Study

In the following section, we give a brief introduction of the human brain and the lobes present in it which are responsible for emotion and also about the characteristics of EEG signals.

### 2.1. Human Brain

**Figure 2:**
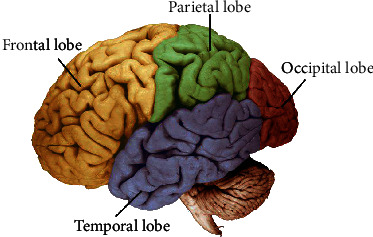
Sectional part of human brain [13].

The brain is the command center for the human nervous system. It is responsible for all of the body's activities. It receives sensory input from the many sensory receptors. Then, it processes, interprets, and decides what actions should be taken. It also sends the output to the muscles. The brain has three main parts viz., cerebellum, brain stem, and cerebrum. The cerebellum accepts data from the many sensory receptors, the spinal cord, and other parts of the brain and then manages motor movements. It also involved involuntary movements such as balance, posture, coordination, and speech, governing smooth muscular function. The brain stem manages the movement of messages between the brain and the other parts of the body. The basic body activities like breathing, swallowing, heart rate, blood pressure, and consciousness are managed by stem. On the other hand, the cerebrum is the largest part of the human brain, and it is further divided into hemispheres viz., left and right. Each hemisphere has four lobes namely, frontal, temporal, occipital, and parietal as in Figure 2. The cerebrum is mainly responsible for the action and thought. The various regions of cerebrum control various actions as described below:

- The frontal region is responsible for the movement of the body, speech, concentration, processing memories from the limbic region, emotional reaction, problem-solving, the meaning of words, personality, and planning. In the frontal lobe region, neurons of dopamine-sensitive type are located which help with the feelings of attention, memory, reward, pleasure, and planning. It contains a part of the limbic region which is responsible for some basic emotions as stated above
- The temporal region is responsible for hearing, emotion, and regulate emotions like hunger, thirst, recognizing faces, and long-term memory which are responsible for storing, forming, and processing visual memory. It contains some part of the limbic region which is responsible for some basic emotion as stated above
- The occipital region is responsible to evaluate the distance, depth, size, helps in color recognition, interprets the visual stimuli, and transmits the visual data to the other regions of the brain and other regions respond to this information
- The parietal region is responsible to feel the sensations of touch, process, taste, and temperature

### 2.2. Emotions

Emotion is a state of feeling and change in both physical and psychological influence of our behavior which we people experience in our daily routine, and it comprises three components namely, abstract understanding, a physiological reaction, and a social or expressive reaction [26, 27]. Emotion has been portrayed as discrete and steady reactions to occasions outside or inner with hugeness for the life form [28]. Emotion plays an important role in human-human communication. Emotion is often interconnected with several factors like mood, personality, temperature, one's circumstances, or relationships with others. The emotional state can be expressed through two dimensions valence and arousal. Valence is the dimension that measures positive or negative effects due to emotion. Arousal is the dimension that measures how soothing or exciting the emotion is, and it is represented in terms of high and low. There are two different aspects of representing emotion. The first aspect shows that essential feelings have advanced through regular choices. Plutchik suggested eight primitive emotions anger, fear, sadness, disgust, surprise, curiosity, acceptance, and joy [29]. In the second aspect, given perception, the feelings are mapped into the valence, arousal, and dominance measurements. Valence goes from extremely positive sentiments to exceptionally negative or unpleasant to joy; excitement goes from states like drowsy to energized; lastly, predominance compares to the quality of the feeling [30]. A considerable lot of our regular exercises rely upon our emotional state. Assessing emotion is the key to understanding people. Consequently, human emotion acknowledgment is vital for the precise classification of human emotion. We will in this manner center around the strategies utilized for recognizing emotion.

As human-human association happens, here come brain-computer interface (BCI) systems. For a technologically invaded life, the brain-computer interaction system plays an important role. As machines impacted our lives from numerous points of view in the previous decade, machines ought to be able to find, translate, get to, and coordinate the emotional states of humans during an interaction. This human-machine association is made simple with BCI frameworks. Nowadays, these BCI frameworks have the capacity for emotion recognition. To have an effective emotion acknowledgment, a huge assortment of strategies like emotion recognition from outward appearances, the pitch of voice, signals from self-ruling sensory system, signals from EEG.

## 3. EEG Signals Database for Affective Computing

A very limited number of EEG signals databases are observed in the literature. This motivates us to develop an EEG signals database for research purposes. This section presents the different protocols that are considered while capturing signals of a human. The detailed description of each protocol is discussed in subsequent sections.

### 3.1. EEG Sensor

EEG is a therapeutic imaging procedure that interprets scalp electrical movement produced by cerebrum structures, i.e., it measures voltage variances coming about because of ionic current streams inside the neurons of the cerebrum. EEG machine used here is EEG traveler braintech 32+ CMEEG-01, which is of 32 channels. This model has many characteristics such as the most friendly used tool-bar, a facility of deleting events and comments, online/offline reformatting of gain, filter, and sweep speed. It also had EEG flash with high-intensity LED with a built-in rechargeable Li-ion battery. It is a highly advanced database management with different searching options. It operates with computer USB power.

**Figure 3:**
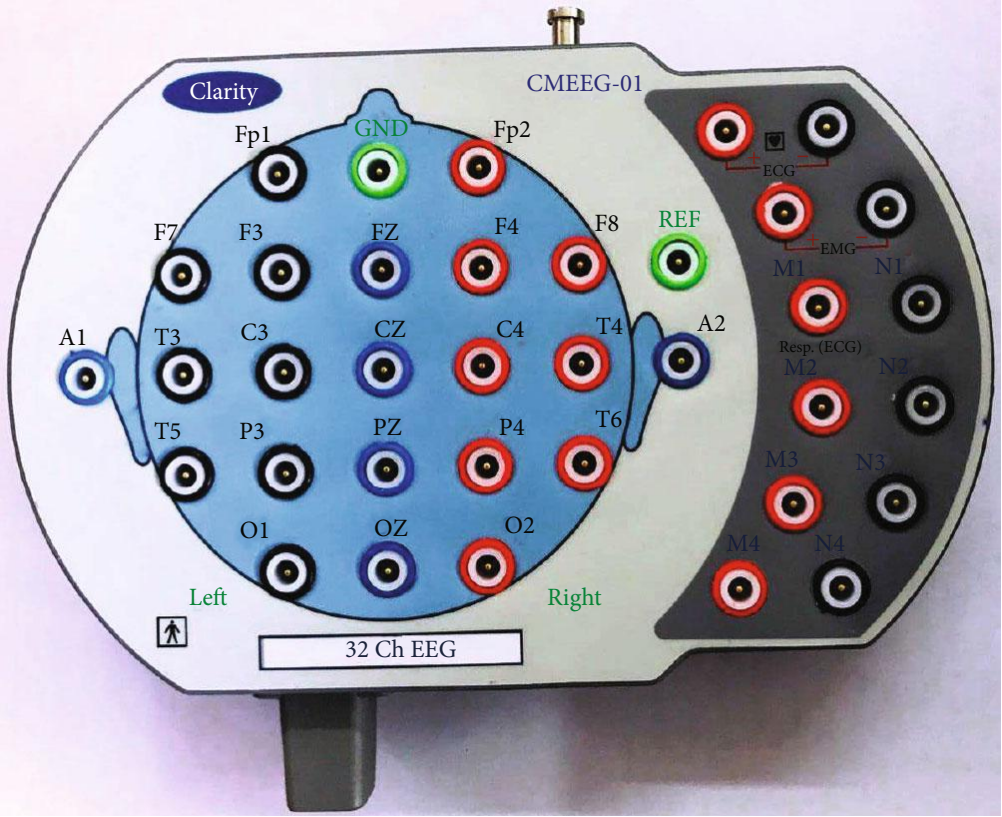
EEG machine for placing electrodes.

Some of the specifications of this EEG traveler are it has 32 input channels and 32 display channels. The frequency band range is from 0.1 Hertz (Hz) to 100 Hz. The maximum impedance limit is 10*MΩ*. The sampling frequency is 1024 Hz for at ADC and 256 Hz for internal storage. It has a high-cut digital filter of the range 0.5 Hz to 100 Hz and low-cut digital filter of the range 0.1 Hz to 7 Hz. There is EEG paste which is used to adhere to the 32 electrodes or channels to the scalp for recording signals. A piece of EEG setup utilized for recording appears in Figure 3.

### 3.2. EEG Signals

EEG signals are principally used to track the action of the human brain utilizing electrical signals. The identification of emotional changes is normally made simple through EEG signals. Along these lines, we have picked this EEG flag-based approach for classification of human emotion to comprehend the essential feelings like happy, fear, sad, and neutral. The EEG recording is carried out with the assistance of the electrodes that are associated with the CLARITY EEG setup. These electrodes are little and level metallic discs that are joined to the scalp utilizing EEG gel/paste. For each action of the human body, cerebrum cells communicate with each other and these exercises of the brain will be recorded in a computer with the assistance of electrodes, and it is estimated in small scale voltage contrast. Voltage contrasts estimated at electrodes are little so they are digitized send to enhancer, and we can see the amplified data in terms of voltage. The EEG signals are helpful for doctors to diagnose disorders like a brain tumor, head injury, and encephalitis. This EEG signal is additionally used to measure the bloodstream level in the cerebrum all through the careful procedure and to screen the brain activity of the individual in unconsciousness/coma. EEG is considered a protected technique. It will not cause any distress or any sensation. It is not ok for a person who is experiencing seizure issue or epilepsy, since it might cause seizure during the procedure as blazing of light on the face, deep breathing is associated with the procedure. It might cause different issues relying upon the medical state of the person experiencing EEG recording.

### 3.3. Brainwaves

Generally, five types of electrical patterns or brain waves have been observed simultaneously by the researchers across the cortex throughout a day in our awaking state or even when we are asleep. These waves in order of lowest frequency to highest are as follows: delta (0-4 Hz), theta (4-7 Hz), alpha (8-13 Hz), beta (13-30 Hz), and gamma (30-63 Hz). It is observed that the range of these waves fluctuates a few Hz among various studies. These waves jointly perform mental functioning effectively and efficiently. However, each brain wave has a unique role. One particular brain wave will be dominant at a time while performing a particular task, and it depends on the state of cognizance that a person is in. If one of the five types of brain waves is either over synthesized and/or under synthesized in our brain, it can cause problems. The detail description of each of the useful brain wave is as follows:

- Delta waves are related to the deepest levels of pleasure and therapeutic rest, healing sleep. These are the slowest captured brain waves in people. Generally, higher levels are noticed in infants and kids. These are produced in a lesser amount as one grows old even during deep sleep. These are also observed too many of our unconscious bodily functions such as regulating heartbeat and digestion. Adequate levels of delta waves help to feel jubilant after we wake up refreshed. However, irregular delta activity may hamper normal learning ability or maintaining awareness
- Theta waves are linked in daydreaming and sleep. These are linked to us experiencing and feeling deep and raw emotions. However, higher levels of theta activity may result in an episode of depression and may make people “highly suggestible” according to the fact that they are in a deeply relaxed, semihypnotic state. These waves help to improve our intuition, creativity, and make us feel more natural. It is also involved in therapeutic rest. The range of these waves is very helpful until not generated in excess during waking
- Alpha waves establish a “frequency bridge” between our cognizant reasoning (beta) and intuitive (theta) mind. These are known to enable quiet us to down and advance sentiments of deeper relaxation and substance. Beta waves assume a functioning part in arranging coordination and correspondence and do not happen until three years of age in person. Higher levels of beta activity and lower levels of alpha activity may result in an emotional state called “alpha blocking.”
- Beta waves are the low-amplitude high-frequency brain waves that are generally found in an awake person. These are produced when a person is incognizant in states like cognitive reasoning, calculation, reading, speaking, or thinking. Higher levels of beta waves may result in stress and anxiety. The beta activity of a person will increase when he or she drinks a stimulant called caffeine that is found especially in tea or coffee
- In the area of neuroscience, a more recent discovery of brainwaves is gamma waves. Researchers are constantly exploring to know more about gamma waves. They have examined a strong relation between meditation and gamma waves. In addition to a healthy cognitive task, these perform more complex tasks. Gamma waves are important for learning, memory, and information processing. It is thought that the 40 Hz gamma wave is important for the binding of our senses in regard to perception and are involved in learning new information. Lower levels of gamma activity are observed in people with mentally challenged

### 3.4. EEG Electrode Positioning

**Figure 4:**
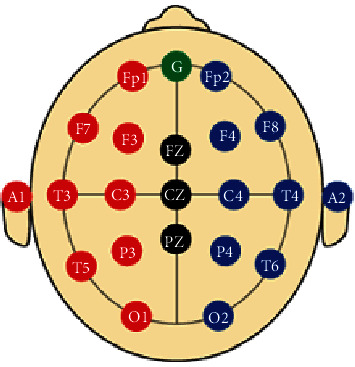
The international 10/20 system for electrode positioning.

The recordings of the EEG signals are performed according to the international standard 10/20 electrode system [31] as shown in Figure 4. Figure 4 depicts the 10/20 international standard electrode system in which the distance between any two adjoining electrodes can be either 10% or 20% of the total front to back or right to the left of the skull. This 10/20 system is based on the connection between the position of the electrode and base zone of the cerebral cortex. Every electrode is demonstrated with a letter and a number which is utilized to recognize the lobe and hemisphere where it must be placed (F, T, P, C, and O letters alludes to frontal, temporal, parietal, central, and occipital lobes separately). There are numbers from 1 to 8 given to the electrodes among which 1, 3, 5, and 7 stand for the electrodes to be placed on the left hemisphere and 2, 4, 6, and 8 stand for the electrodes to be placed on the right hemisphere. “Z” refers to the electrode that is to be placed on the midline. Besides these electrodes, we also have ground and reference electrodes. Four anatomical reference points on head are utilized for the outright position of electrodes specifically nasion, inion, and both preauricular focuses. Nasion is the middle of forehead and nose, inion is the point from the posterior of the head which is the lowest of the skull, and preauricular focuses are those which are front to ear [32].

### 3.5. Impedance Check

**Figure 5:**
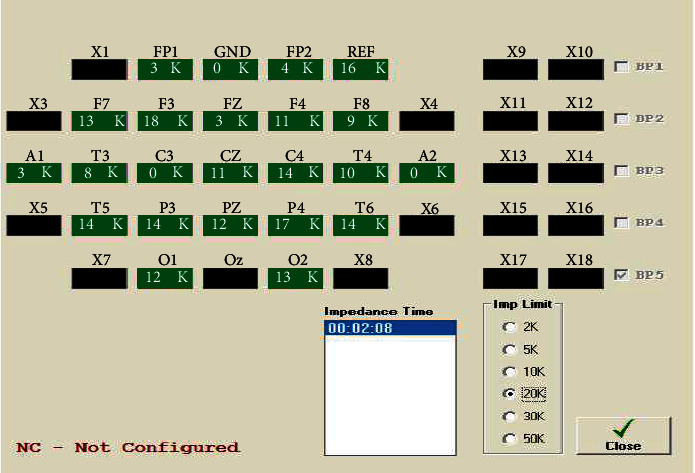
Impedance check.

After placing all the electrodes in their desired positions, we should check the impedance, which is whether all the electrodes are connected properly or not. If not, we should again check those electrodes which are not connected properly and connect them. The impedance is verified in the software which is provided with the CLARITY EEG machine. During the recording procedure, the impedance of the electrodes should not exceed the maximum impedance limit. The impedance corresponding to each electrode is shown in Figure 5. If the limit exceeds the maximum impedance level, color shown in the impedance check option of the software used to record the EEG signals changes from green to red. During this impedance check, the recording is stopped.

### 3.6. Stimuli

**Figure 6:**
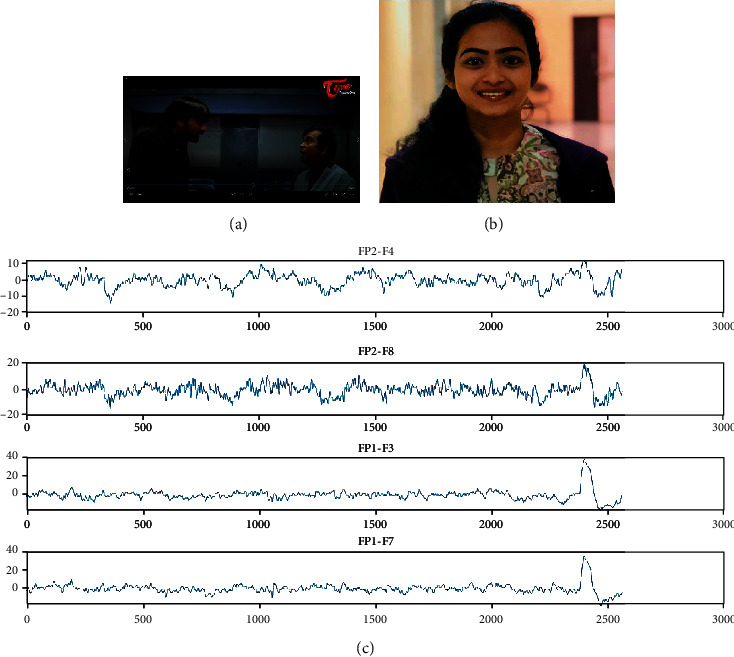
(a) The snapshot of happy stimuli (b) reaction of a subject (c) captured EEG signals (*x*-axis and *y*-axis show time in second and amplitude in *μV*, respectively).

**Figure 7:**
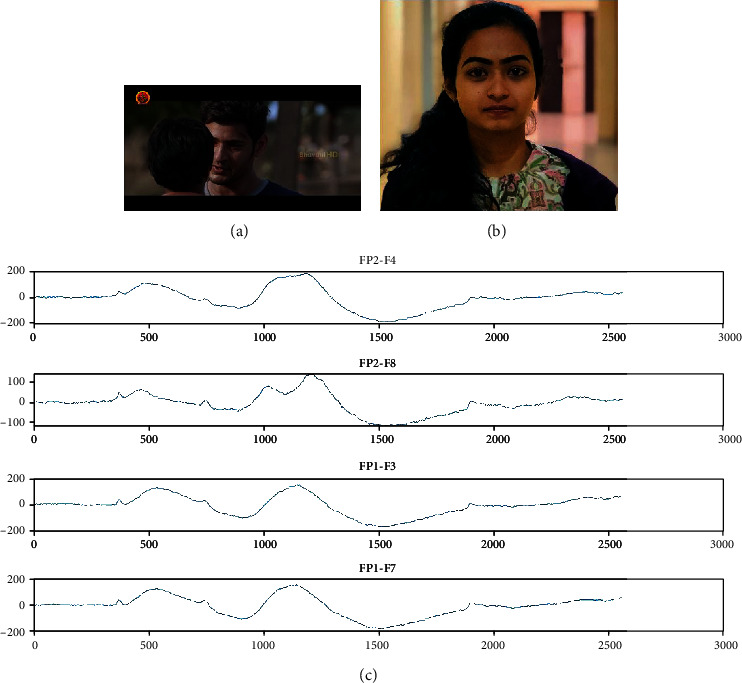
(a) The snapshot of sad stimuli (b) reaction of a subject (c) captured EEG signals (*x*-axis and *y*-axis show time in second and amplitude in *μV*, respectively).

**Figure 8:**
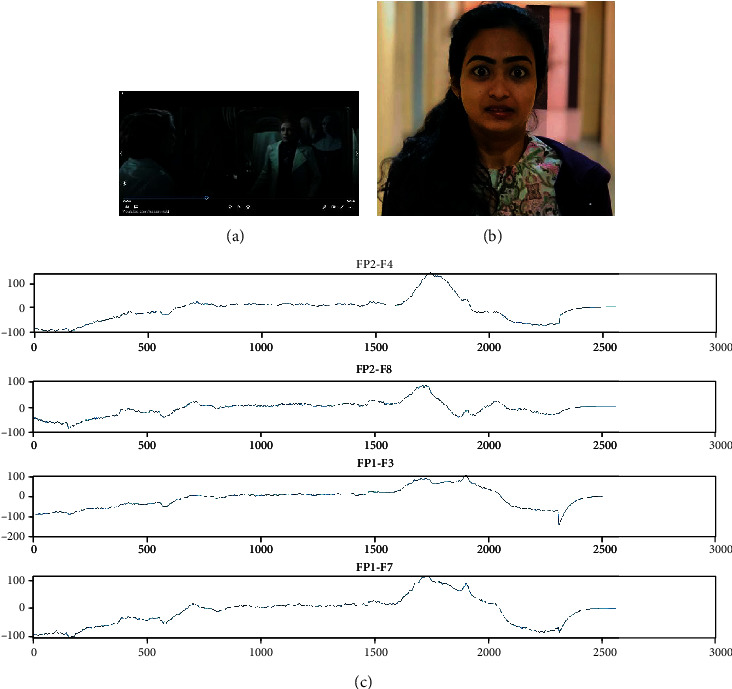
(a) The snapshot of fear stimuli (b) reaction of a subject (c) captured EEG signals (*x*-axis and *y*-axis show time in second and amplitude in *μV*, respectively).

**Figure 9:**
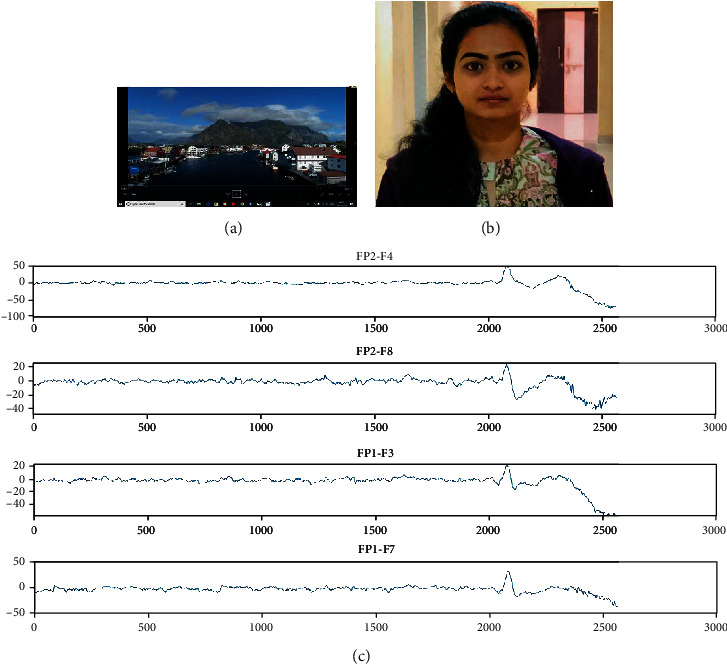
(a) The snapshot of neutral stimuli (b) reaction of a subject (c) captured EEG signals (x-axis and y-axis show time in second and amplitude in *μV*, respectively).

The stimuli selected in this experiment through various steps. First, we should consider the different types of emotion, and among them, we should select some which we want for classification. Here, four emotions namely, sad, happy, fear, and neutral are considered. Total stimuli have 12 videos, which means each emotion has 3 videos. Each video is of approximately 150 seconds which is 2 minutes and 30 seconds [33]. After watching every video, the person should relax for 15-20 seconds for neutralizing the emotion that he had felt during that period. During relaxing for a certain period, the person should close his/her eyes and relax to avoid the surroundings and prevent disturbances around them. The signals have been recorded in a close room with no lights and room temperature is 25-30 degrees Celsius. We have selected the stimuli of our required emotion from featured films based on the criteria like comedy scenes for happy emotion and emotional scenes for sad motion and scary scenes for fear emotion and neutral scenes for neutral emotion. There should be no noise or disturbance around the one who is watching the stimuli because, if any disturbance has taken place, the person might get deviated from the emotion, and the signals during that period of disturbance will be useless. The snapshot of happy stimuli, the reaction of a subject while watching that stimuli, and the captured EEG signals of four channels namely, FP2-F4, FP2-F8, FP1-F3, and FP1-F7 are shown in Figure 6.  Figure 7 shows the snapshot of sad stimuli, the subject's reaction, and the acquired EEG signals, respectively. The snapshot of fear stimuli, the reaction of a subject while watching that stimuli, and the captured EEG signals are shown in Figure 8.  Figure 9 illustrates the snapshot of neutral stimuli, the subject's reaction, and the acquired EEG signals, respectively. The horizontal and vertical axes of Figures 6(c), 7(c), 8(c), and 9(c) are mean sample sizes and amplitudes of EEG signal, respectively.

### 3.7. Pattern Matching

These 12 videos are divided into 3 patterns in which each pattern consists of 4 videos of a different emotion. As the person after watching the first round of the pattern, he needs to match the videos according to the emotion provided on the left side. We should take care of creating these patterns while arranging the videos, because no pattern should be the same as of the previous one.

**Figure 10:**
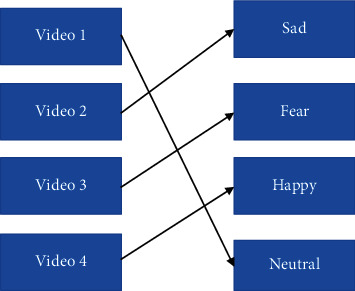
Pattern 1 for watching stimuli.

*Pattern 1*: neutral–sad–fear–happy (Figure 10).

**Figure 11:**
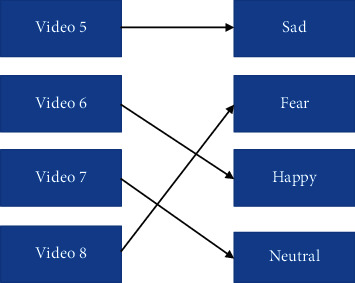
Pattern 2 for watching stimuli.

*Pattern 2*: sad–happy–neutral–fear (Figure 11).

**Figure 12:**
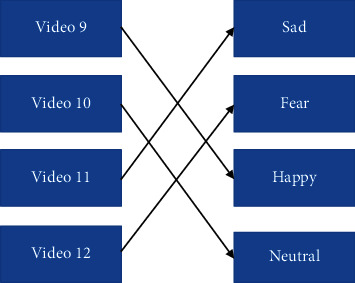
Pattern 3 for watching stimuli.

*Pattern 3*: happy–neutral–sad–fear (Figure 12).

This is because of the person who is matching the pattern should remember the four videos and match the pattern as to how he felt. For better results, the patterns should be varied. It is because if we change pattern we can make sure that the person who is matching the pattern is matching as how he felt rather than remembering the previous pattern and matching as it is in the new one.

### 3.8. Database Summary

No. of videos: 12. Video duration: 150 seconds (approximately). Selection method: manually. Total no. of participants: 44 (23 male + 21 female).

## 4. Materials and Methods

**Figure 13:**
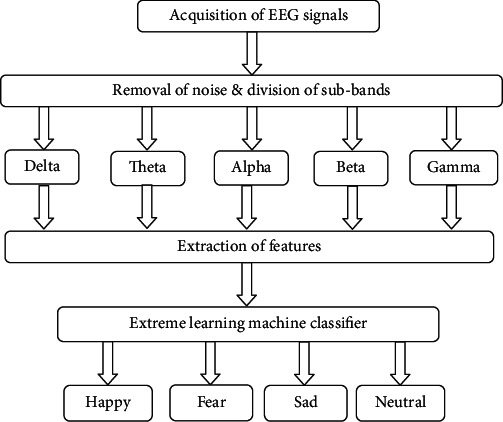
Block diagram of the proposed methodology.

A schematic block diagram of the proposed system for classifying emotion is displayed in Figure 13. The proposed method includes EEG signal acquisition, removal of noise and division of subbands, extraction of features, and classification. EEG signals acquisition procedure is presented in Section 3. The rest of the steps would be described in the subsequent sections.

### 4.1. Discrete Wavelet Transform

**Figure 14:**
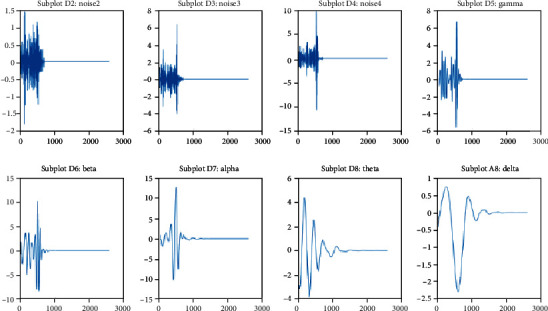
The different subbands of a randomly selected channel from neutral emotion produced by “db8” DWT (*x*-axis and *y*-axis show time in second and amplitude in *μV*, respectively).

It is clear from Section 3.3 that EEG signals consist of mainly five types of brain waves. However, EEG signals also include noise. It is an unwanted signal that corrupts the signal of interest. So, the noise has to be removed from the raw signals to extract useful information. Thus, the first step in the processing of EEG signals is the filtering of raw signals. EEG signal is nonstationary, and multiresolution DWT is a widely used decomposition method for eliminating noise from a nonstationary signal [26]. The frequency of the EEG signal that we have captured is 256 Hz. Daubechies 8 DWT (db8) is adopted here to decompose raw EEG signal into detail coefficients and approximation coefficients up to 6^*th*^ level. So, a total of 6 detail subbands (D1 to D6) and 1 approximation subband (A6) would be generated. These subbands are also called waves. Here, D1, D2, D3, D4, D5, D6, and A6 subbands are known as noise (125-256 Hz), noise (63-125 Hz), gamma (30-63 Hz), beta (13-30 Hz), alpha (8-13 Hz), theta (4-7 Hz), and delta (0-4 Hz), respectively. The various subbands obtained using “db8” DWT of a randomly selected channel from neutral emotion are shown in Figure 14.

### 4.2. Extraction of Features

The extraction of features is a process of unveiling hidden characteristics from the input signal. In other words, a set of features can represent an input signal. Moreover, this feature set illustrates a specific behavior or pattern depicted by the original input signal. Every signal is normalized to zero mean and unit variance. Ten popularly used features are extracted from each subband for emotion classification considered in this work, which is as follows:

(i) Average amplitude change
 Average amplitude change is the mean value of the changes between two consecutive sample values. The value of AAC is estimated using Eq. (1).
  (1) $$\begin{array}{c} f_{1}=\frac{1}{C}\overset{C-1}{\underset{i=1}{\sum }}\;\left|s_{i+1}-s_{i}\right|, \end{array}$$
  where *s*_*i*_ is the *i*^*th*^ sampling point of an EEG signal and C is the total number of sample points. In our case, the value of C is 2560 because the sampling frequency of the device used is 256 Hz, and the duration of the signal is 10 s. It is also referred to as difference absolute mean value.
(ii) Activity
 Activity is the measure of the mean power which is calculated by Eq. (2). It also represents the activeness of the signal. It also represents the variance of the EEG signal.
  (2) $$\begin{array}{c} f_{2}=\sigma_{a}^{2}, \end{array}$$
  where *σ*_*a*_^2^ represents the variance of an EEG signal.
(iii) Absolute square root sum
 The absolute square root sum is calculated using Eq. (3) which is the sum of the square root of each sample point in a signal.
  (3) $$\begin{array}{c} f_{3}=\overset{C}{\underset{i=1}{\sum }}\;\sqrt{s_{i}}, \end{array}$$
  where *C* is the total number of sample points.
(iv) Clearance factor
 The clearance factor is calculated through Eq. (4), and it is a ratio of the peak value of a signal to the square of the average of the square root of the absolute value of the EEG signal.
  (4) $$\begin{array}{c} f_{4}=\left(\frac{peak-value}{{\left(1/C\sum_{i=1}^{C}\;\sqrt{\;|\;s_{i}\;|\;}\right)}^{2}}\right), \end{array}$$
  where peak value is obtained using (1/2)(max(*s*_*i*_) − min(*s*_*i*_)).
(v) Root mean square
 Root mean square (RMS) is defined as the square root of the average of the square of each sample point of a signal which is estimated using Eq. (5).
  (5) $$\begin{array}{c} f_{5}=\sqrt{\frac{1}{C}\overset{C}{\underset{i=1}{\sum }}\;{\left(s_{i}\right)}^{2}}. \end{array}$$
(vi) Crest factor
 Crest factor is the ratio of the peak value of the signal to the RMS value, and it is found using Eq. (6).
  (6) $$\begin{array}{c} f_{6}=\left(\frac{peak-value}{RMS}\right). \end{array}$$
(vii) Shape factor
 The shape factor is the ratio of RMS value to the average of the absolute value of the signal which is computed by Eq. (7).
  (7) $$\begin{array}{c} f_{7}=\left(\frac{RMS}{1/C\sum_{i=1}^{C}\;\left|s_{i}\right|}\right). \end{array}$$
(viii) Log detector
 The nonlinear detector is defined as Eq. (8).
  (8) $$\begin{array}{c} f_{8}=e^{1/C\sum_{i=1}^{C}\log\left(s_{i}\right)}. \end{array}$$
(ix) Mobility
 Mobility is approximated from Eq. (9) which is defined as the square root of the ratio of the variance of the first derivative of signal to the variance of the signal.
  (9) $$\begin{array}{c} f_{9}=\frac{\sigma_{d}}{\sigma_{a}}. \end{array}$$
(x) Absolute sum
 Absolute sum is defined by Eq. (10).
  (10) $$\begin{array}{c} AS=\overset{C}{\underset{i=1}{\sum }}\;\left|s_{i}\right|. \end{array}$$
 Every machine learning algorithm is associated with some objective functions. Sometimes, these objective functions may not work properly without normalization, because the range of values of raw data varies greater than usual. For example, most of the classifiers estimate the distance between two points by the Euclidean norm. If one of the features or attributes has a wide range of values, the distance will be governed by this specific feature. Thus, the range of all features should be normalized so that each feature contributes approximately proportionately to the final distance [34]. So, normalization is done on extracted features also.

### 4.3. Extreme Learning Machine Classifier

The ELM is an efficient single-layer feedforward neural networks [35]. It is mainly considered as a classifier, regressor, and cluster analysis. However, it is adopted here for emotion classification because of the better generalization, controllability and robustness, and fast learning rate. It can solve the overfitting problem as compared to other conventional neural networks. It uses the empirical risk minimization theory. Moreover, it averts local minimization and multiple iterations. Furthermore, the learning process of the ELM takes a single iteration only. Finally, tune is not required for updating the parameters of hidden nodes. These nodes can be acquired from their forefathers without any modification or can be randomly assigned. Normally, the outcome weights of hidden nodes are gained in a single step. Let us consider an ELM with a single hidden layer and the output function of the *j*^*th*^ hidden node is *m*_*j*_(*x*) = *f*(*α*_*j*_, *β*_*j*_, *x*) = 1/1 + exp(−(*α*_*j*_*x* + *β*_*j*_)), where f(.) is a sigmoid function and *α*_*j*_ and *β*_*j*_ are the parameters of the *j*^*th*^ hidden node. The output function of the ELM for single-layer feedforward network with *k* hidden nodes is expressed by Eq. (11).

(11) $$\begin{array}{c} g_{k}\left(x\right)=\gamma_{1}m_{1}\left(x\right)+\gamma_{2}m_{2}\left(x\right)+\cdots +\gamma_{j}m_{j}\left(x\right)+\cdots +\gamma_{k}m_{k}\left(x\right), \end{array}$$

where *γ*_*j*_ is the output weight of the *j*^*th*^ hidden node. The outcome of the hidden layer is estimated using Eq. (12).

(12) $$\begin{array}{c} m\left(x\right)=\left[f\left(m_{j}\left(x\right),\cdots ,m_{k}\left(x\right)\right)\right]. \end{array}$$

The hidden layer yield matrix *M* of ELM is expressed by Eq. (13), when the number of training samples is *N*.

(13) $$\begin{array}{c} M=\left[\begin{array}{c} m\left(x_{1}\right)\\ \vdots \\ m\left(x_{N}\right) \end{array}\right]=\left[\begin{array}{ccc} f\left(\alpha_{1},\beta_{1},x_{1}\right) & \cdots & f\left(\alpha_{l},\beta_{l},x_{1}\right)\\ \vdots & \vdots & \vdots \\ f\left(\alpha_{1},\beta_{1},x_{N}\right) & \cdots & f\left(\alpha_{l},\beta_{l},x_{N}\right) \end{array}\right]. \end{array}$$

The training data target vector is represented by Eq. (13).

(14) $$\begin{array}{c} L=\left[\begin{array}{c} l_{1}\\ \vdots \\ l_{N} \end{array}\right]. \end{array}$$

The objective function of ELM is shown in Eq. (14).

(15) $$\begin{array}{c} {\left\|\gamma \right\|}_{p}^{\sigma_{1}}+C{\left\|M\gamma -L\right\|}_{q}^{\sigma_{2}}, \end{array}$$

where *σ*_1_, *σ*_2_ > 0 and *p*, *q* = 0, 0.5, 1, 2, ⋯ The basic form of ELM algorithm considered in this work is as follows:

(16) $$\begin{array}{c} \hat{Y}=W^{2}\sigma \left(W^{1}x\right), \end{array}$$

where *W*^1^ and *W*^2^ are the two matrices used to denote weights of input to hidden layer and hidden to output layer, respectively, and *σ* is the sigmoid activation function. The steps of the ELM algorithm are as follows:

(17) $$\begin{array}{c} W^{2}=\sigma {\left(W^{1}X\right)}^{+}Y, \end{array}$$

where *X*, *Y*, and + denote feature matrix, response variables, and pseudoinverse, respectively. Scholars use the ELM due to the property of low computational complexity. Most training can be accomplished in minutes, and even in some cases, the ELM takes seconds to complete the training process. The complexity of the ELM can be represented by *O*(*nd*), where *n* and *d* denote input weights and biases, respectively [36].

**Step 1 .**

*W* ^1^ is filled by some random values.

**Step 2 .**

*W* ^2^ is estimated as per Eq. (17).

## 5. Experimental Results and Discussions

All the programs are implemented in Matlab 2017a, and these programs are executed on a laptop of the following specification: Intel(R) Core(TM) i5 5200U CPU @ 2.20 GHz, 4 GB RAM, 64-bit Windows Operating System.

At the very first step, DWT takes noisy EEG signals as inputs and produces noise-free EEG signals in the form of five subbands as outputs. Every subband is normalized to zero mean and unit variance. Ten features are extracted from these subbands in the second step. Then, all the features are normalized in the range of 0 and 1. In this study, 44 volunteers and their four emotions are considered for classification. Three videos are captured for each emotion. Ten pages of information for each video are stored while capturing videos. Four channels namely, FP2-F4, FP2-F8, FP1-F3, and FP1-F7 are selected out of 32 channels as these channels are placed in the frontal region of the head, and the frontal region is mainly responsible for identifying emotion correctly. Each channel has 5 subbands after noise removal. So, we have (44 × 3 × 10 × 5 × 4) = 6600 samples altogether for each emotion. It means we get a feature matrix of size 26400 × 10, where 26400 is the sample size and 10 features are there for each sample. Three experiments are performed in this study. In the first experiment, channel-wise classification is carried out to know which channel is more responsible for emotion recognition. Several samples of each channel would be equal to (44 × 3 × 10 × 5 × 4) = 6600, and each channel will have 10 features. The feature matrix is divided into two sets namely, training and testing sets. The former one has 70% of total samples, and the rest of the samples are part of the testing set. Training samples and their class labels are fed into ELM for building a trained model. Finally, the test sample would be provided one after another as input and predicts its class label as output with the help of the trained model. Five measures namely, accuracy, specificity, sensitivity/recall, precision, and f1-score [37–39] are considered in this study for measuring the emotion recognition performance of each channel. The obtained value of each measure is reported in Table 1. It is clear from Table 1 that the performance of the first channel, i.e., FP1-F7 is better than the other three channels as the values of accuracy, sensitivity, specificity, precision, and f1-score are 94.72, 98.24, 94.72, 94.71, and 94.71, respectively, which are the highest among the four channels.

Subband wise emotion classification is performed in the second experiment. The number of samples of each subband would be equal to 44 × 3 × 10 × 4 = 5280. So, the size of feature matrix would be 5280 × 10. Then, 70% of the total samples are allocated for training. The remaining 30% samples are referred to as the testing set. ELM is trained by the training samples and their class labels. Test set is used then to predict the class labels of the testing samples. Accuracy is computed based on actual and predicted classes, and subband wise performance accuracy is reported in Table 2.

It is observed from Table 2 that the gamma subband provides the highest overall accuracy among all the subbands. Here, no emotion is considered separately. So, in the final experiment, only features are extracted from the gamma subband of the FP2-F4 channel. The number of samples would be equal to 44 × 3 × 10 = 1320 for each emotion. Again, the same procedure is adopted for classifying emotion by ELM. Emotion-wise classification performances are shown in Table 3.  Table 4 reports the classification performance that is achieved with the help of features extracted from the gamma subband of the FP2-F8 channel. Similarly, the recognition performances are reported in Tables 5 and 6 only when features are obtained on gamma subband of the FP1-F7 channel.

## 6. Conclusion

Classification of human emotion using EEG signals is the best possible method, because it provides the actual neurophysiology of brain states. In this work, DWT decomposes each EEG signal into subbands which are further used for extraction of features from that subbands. The channel-wise extracted features are fed as inputs to the ELM classifier for the classification of happy, fear, sad, and neutral emotion. This method provides better results using a single EEG channel. The single-channel-based emotion classification ability of the proposed method shows its adroitness for the practical applications of emotion classification in BCI systems. Recent state-of-the-art methods would also be considered to compare our proposed method. Deep learning convolution neural network may be employed to denoise the EEG signals followed by classification emotions. Here, we mainly concentrated to capture EEG signals database. We are also planning to consider more number of subjects to extend the database. We will focus to make a multimodality based BCI system for emotion recognition using face biometrics and physiological signals such as EEG signals, blood pressure, respiratory rate, body temperature, and galvanic skin response in near future.

## Figures and Tables

**Table 1:** Channel-wise performance comparison in percentage.

	FP2-F4	FP2-F8	FP1-F3	FP1-F7
Accuracy	90.85	91.23	94.01	94.72
Specificity	96.95	97.07	98.03	98.24
Sensitivity	90.85	91.23	94.01	94.72
Precision	90.82	91.02	94.09	94.71
F1-score	90.82	91.20	94.09	94.71

**Table 2:** Subband wise performance comparison for different channels.

Channels	Alpha	Beta	Gamma	Theta	Delta
FP2-F4	86.53	88.01	90.85	73.86	84.05
FP2-F8	85.79	88.21	91.23	80.67	85.95
FP1-F3	92.34	91.09	94.01	75.93	83.73
FP1-F7	94.70	94.23	94.72	76.44	86.16

**Table 3:** Emotions classification performance using gamma subband of FP2-F4 channel.

	Happy	Sad	Neutral	Fear
Accuracy	95.71	93.63	94.15	97.68
Specificity	96.83	96.01	96.29	98.13
Sensitivity	92.59	86.76	88.00	96.48
Precision	91.26	88.30	89.17	94.98
F1-score	91.92	87.52	88.58	95.72

**Table 4:** Emotions classification performance using gamma subband of FP2-F8 channel.

	Happy	Sad	Neutral	Fear
Accuracy	96.07	94.85	93.92	96.95
Specificity	97.42	96.85	95.54	98.38
Sensitivity	92.31	90.02	89.08	93.14
Precision	92.75	90.18	87.01	95.57
F1-score	92.53	90.10	88.03	94.34

**Table 5:** Emotions classification performance using FP1-F3 channel and gamma subband.

	Happy	Sad	Neutral	Fear
Accuracy	98.33	95.25	94.72	99.74
Specificity	98.72	96.77	96.59	99.75
Sensitivity	97.22	90.74	89.17	99.72
Precision	96.42	90.50	89.78	99.30
F1-score	96.82	90.62	89.47	99.51

**Table 6:** Emotions classification performance using FP1-F7 channel and gamma subband.

	Happy	Sad	Neutral	Fear
Accuracy	97.95	95.86	95.75	99.69
Specificity	96.85	97.56	97.16	99.87
Sensitivity	97.42	90.85	91.59	99.18
Precision	94.95	92.66	91.59	99.61
F1-score	96.17	91.75	91.59	99.39

## Data Availability

The “EEG signals related to emotions” data used to support the findings of this study are currently under embargo, while the research findings are commercialized. Requests for data, 6 months after publication of this article, will be considered by the corresponding author under license agreement.
